# Sexual Dimorphism in the Association of Serum Retinol-Binding Protein-4 With Long-Term Dynamic Metabolic Profiles in Non-Diabetes

**DOI:** 10.3389/fendo.2022.880467

**Published:** 2022-05-11

**Authors:** Jiali Xiang, Huajie Dai, Yanan Hou, Qi Wang, Tiange Wang, Mian Li, Zhiyun Zhao, Jieli Lu, Meng Dai, Di Zhang, Yu Xu, Guang Ning, Weiqing Wang, Jiqiu Wang, Yufang Bi, Min Xu

**Affiliations:** ^1^ Department of Endocrine and Metabolic Diseases, Shanghai Institute of Endocrine and Metabolic Diseases, Ruijin Hospital, Shanghai Jiao Tong University School of Medicine, Shanghai, China; ^2^ Shanghai National Clinical Research Center for Metabolic Diseases, Key Laboratory for Endocrine and Metabolic Diseases of the National Health Commission of the PR China, Shanghai Key Laboratory for Endocrine Tumor, State Key Laboratory of Medical Genomics, Ruijin Hospital, Shanghai Jiao Tong University School of Medicine, Shanghai, China

**Keywords:** retinol-binding protein-4, long-term follow-up, dynamic change, adiposity, metabolic profile

## Abstract

**Objectives:**

We aimed to investigate the association of circulating retinol-binding protein-4 (RBP4) levels with long-term cardiometabolic risk profiles and whether sex disparity mattered.

**Methods:**

We included 784 non-diabetic participants aged 40 years and above from a well-defined community-based cohort at baseline in 2005 and they were invited to attend the on-site follow-up examination for two consecutive times with 3-year intervals in 2008 and 2011, respectively. Serum RBP4 was measured at baseline, and the anthropometry and biochemical measurements were performed at each visit. Generalized estimating equation models were used to assess the association of serum RBP4 levels with the dynamic changes in adiposity and glucolipid profile.

**Results:**

Based on all the baseline and the 3- and 6-year follow-up data, baseline serum RBP4 levels (each 1-unit of log_10_RBP4) were significantly associated with waist circumference [β=3.12, 95% confidence interval (CI) (0.77, 5.47), P=0.01], fasting, and 2-h post-loading glucose [β=0.26 (0.05, 0.47), P=0.02, and 1.70 (1.29, 2.12), P< 0.0001], serum triglycerides [β=0.75, 95% CI (0.54, 0.96), P< 0.0001], total cholesterol [β=0.47, 95% CI [0.23 0.70], P<0.0001), and marginally with body mass index (β=0.97, 95% CI (0.02, 1.93), P=0.046], in total participants, after adjusting potential confounders. The association of RBP4 with 2-h post-loading glucose was stronger in women than that in men [β=1.99, 95% CI (1.49, 2.50) vs. 0.61 (-0.14, 1.36), P for interaction=0.001]. The analysis of change in Z-score of cardiometabolic profiles corresponding to each 1-unit increment in log_10_RBP4 showed consistent results.

**Conclusions:**

Higher RBP4 levels are associated with longitudinal increase in adiposity and deteriorated glucolipid profile defined by repeated measurements. The associations differ in sex regarding to the 2-h post-loading glucose.

## Introduction

Retinol-binding protein 4 (RBP4), a retinol transporter in circulation belonging to the lipocalins family (1), has been reported to be associated with type 2 diabetes, obesity, insulin resistance, and other cardiometabolic disorders (2–5). RBP4 levels are higher in obese and/or diabetic individuals in cross-sectional studies (2, 3), and moderate weight reduction can lower serum RBP4 levels in nondiabetic subjects (3). In our previous study, we also found that serum RBP4 was associated with a higher risk for impaired glucose regulation (5). Several prospective cohort studies reported that serum RBP4 levels were associated with increased risk of diabetes and cardiovascular mortality in men with type 2 diabetes (6, 7). However, the results were not consistent. RBP4 concentrations are not increased in children as they are in obese adults with long-standing severe insulin resistance and type 2 diabetes (8). Recent research reported a U-shaped relationship between serum RBP4 levels and the risk of incident type 2 diabetes in subjects with prediabetes (9). The long-term effect of RBP4 on obesity, glucose, and other cardiometabolic risk needs to be further clarified.

Sexual dimorphisms in cardiometabolic disease features including prevalence, progression, and outcome were reported in the epidemiological and clinical setting. Recently, sex difference has been reported to play a pivotal role in the pathogenesis of several cardiometabolic disease (10–13). A recent nested case-control study performed in Singapore Chinese has reported a sex-specific association of RBP4 with risk of type 2 diabetes (14). However, whether sex disparities matter in the association of RBP4 levels with metabolic profiles remains underexplored.

In current longitudinal cohort study with metabolic traits repeatedly measured and followed up for two consecutive times with 3-years’ interval, we investigated the association of baseline serum RBP4 with long-term dynamic metabolic profile changes including adiposity, blood glucose, and lipid metabolism. Meanwhile, we lay a particular emphasis on sex disparities in the relationship between baseline RBP4 levels and the metabolic profiles.

## Methods

### Study Participants

The study participants were from a two-step blood glucose survey started at 2004, performed in an urban community of Shanghai. The study protocols were approved by the Institutional Review Board of the Ruijin Hospital, and written informed consent was provided by each participant. The detailed study design and protocols of this community-based cohort have been described in previous literature (5, 15). All local permanent residents aged 40 or older were invited and, in total, 9219 individuals attended the first step of this survey. After excluding those with previously diagnosed diabetes, remaining screened participants were classified into three groups according to capillary glucose concentrations: 7.0 mmol/L or above; 5.6–6.9 mmol/L; or below 5.6 mmol/L. There were 631 people with fasting capillary glucose levels of 7.0 mmol/L or above, 757 people with levels 5.6–6.9 mmol/L, and 909 individuals with levels below 5.6 mmol/L selected for further investigation, which were sex- and age-matched and based on a ratio of 1:1.2:1.44, in consideration of individuals with lower glucose levels might be less likely to participate than those with higher glucose concentrations. A total of 1835 individuals (491 with fasting capillary glucose levels of 7.0 mmol/L or above; 594 with levels at 5.6–6.9 mmol/L; and 750 with levels below 5.6 mmol/L) participated in the second step of the survey, with an overall recall rate of 80%. During the second step survey, a 75-g oral glucose tolerance test (OGTT) was performed, and both overnight fasting and 2 h OGTT venous blood samples were collected.

**Figure 1:**
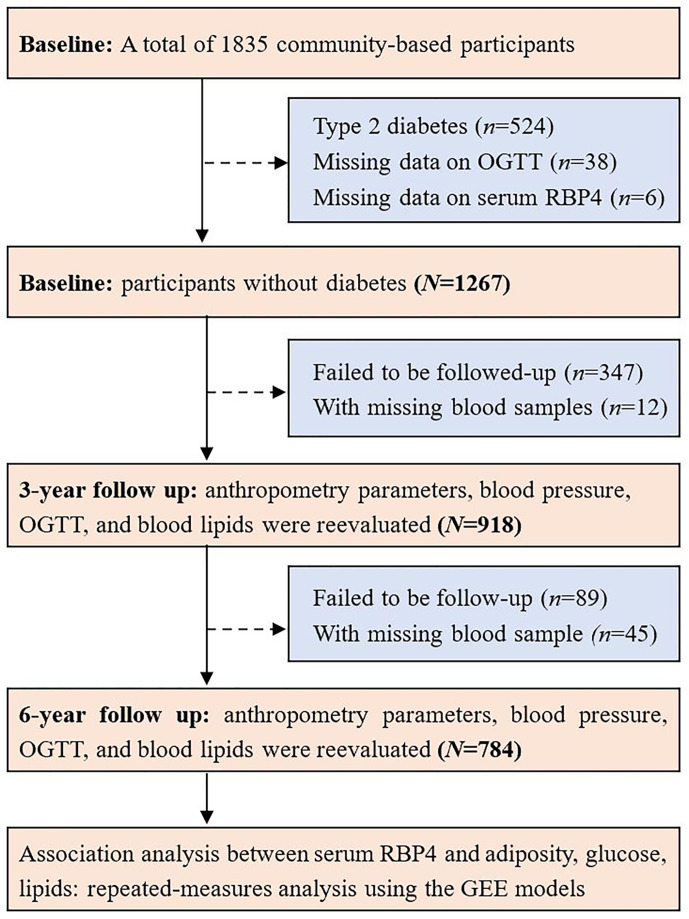
Flow chart of study population.

All the participants were invited to attend the follow-up visits for two consecutive times with a 3-year interval. Similarly, a 75-g OGTT and blood sampling were also performed in the subsequent follow-up visits. There were 1267 participants without diabetes at baseline, 918 and 784 individuals attended the follow-up visit and with blood sampled in 2008 and 2011, respectively. For the present study, 784 individuals without diabetes at baseline and attended the baseline and the two follow-up visits during a period over 6 years were included in the final analysis. A flow chart showing the study procedure is shown in **Figure 1**.

### Data Collection

Information on sociodemographic characteristics, lifestyle, disease, and medication history was collected by questionnaire at baseline and the subsequent visits. Current smokers or drinkers were defined as individuals who smoked or drank regularly in the past 6 months. Anthropometry traits like body height, body weight, and waist and hip circumferences were measured by well-trained examiners, when participants were dressed in light clothes and with bare feet. Body mass index (BMI) was calculated as body weight in kilograms divided by height squared in meters. Blood pressure was measured at the right arm three times consecutively with 1 min intervals after at least 5 min of rest in the seated position, with an automated electronic device (OMRON Model1 Plus; Omron Company, Kyoto, Japan).

### Clinical and Biochemical Measurements

Overnight fasting and 2-h post glucose-load plasma glucose concentrations and fasting serum concentrations of triglycerides, total cholesterol, high-density lipoprotein (HDL) cholesterol and low-density lipoprotein (LDL) cholesterol, uric acid, and creatinine were measured using an automatic analyzer (Beckman CX-7 Biochemical Autoanalyzer, Brea, CA). Serum insulin concentrations were measured by radioimmunoassay (Sangon Company, Shanghai, China). The homeostasis model assessment of insulin resistance (HOMA-IR) was calculated as the following formula (16): HOMA-IR=fasting insulin (uU/L) ×fasting glucose (mmol/L)/22.5. HOMA-β was calculated as: HOMA-β=20×fasting insulin (uU/L)/[(fasting glucose (mmol/L)-3.5].

Serum RBP4 levels were measured at baseline in duplicate by enzyme linked immunosorbent assay developed in-house, using polyclonal and monoclonal antibodies generated against recombinant human RBP4 protein and purified using affinity chromatography (4, 5). The assay system has good precision with both inter-assay variation coefficient and intra-assay variation coefficient below 20%, and it was further cross-validated by Western blotting. The specific steps of serum RBP4 measurement were given previously.

### Statistical Analysis

Continuous variables in normal distribution were reported as means ± SDs, while the skewed ones are presented as medians (interquartile ranges). Categorical variables are presented as frequencies (%). The comparison of the metabolic traits for the three measurements at baseline, 3-year, and 6-year follow-up was conducted by using the analysis of covariance after adjustments for age and sex.

Generalized estimating equations (GEE) model, accounting for correlation over time instead of assuming independence, is often used to analyze longitudinal data (17). The GEE model was used to reveal the associations of baseline serum RBP4 levels and adiposity index and glucolipid profile in total participants. The analysis was adjusted for potential confounders. Model 1, adjusted for age and sex; model 2, further adjusted for BMI, and smoking and drinking habits based on model 1; and model 3, further adjusted for anti-diabetes drug usage in glucose metabolism traits or lipid-lowering drug usage in lipid profile. We also performed the association analysis between baseline RBP4 and the metabolic traits at each examination visit separately and compared the trajectory consistence of the associations.

To further detect whether sex affects the association between serum RBP4 levels and plasma glucolipid profile, we performed a subgroup analysis according to men vs. women and tested the significance of the multiply interaction with sex. Metabolic traits were also standardized to Z score to facilitate comparison of RBP4 effect sizes.

*P* values of less than 0.05 were considered statistically significant for all the analyses. SAS software version 9.4 (SAS Institute, Cary, NC) was used to perform statistical analysis.

## Results

### Characteristics of Participants

**Figure 2:**
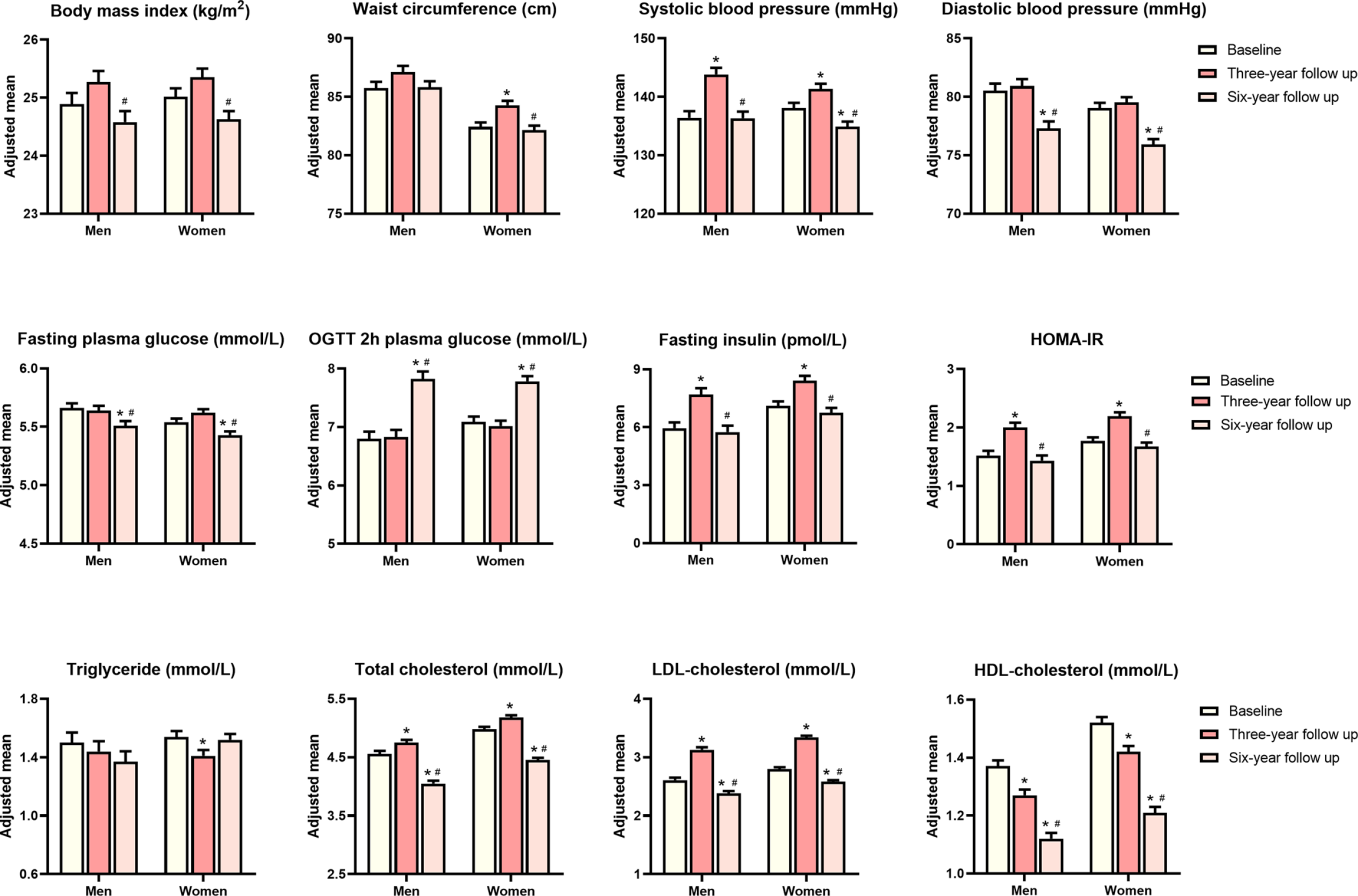
Age adjusted means of metabolic traits at baseline, 3-year follow-up and 6-year follow-up. P values were calculated from analysis of covariance. *P value significant compared with baseline, ^#^P value significant compared with 3-year follow-up.

The general characteristics of 784 participants who attended three visits are summarized in **Table 1**. The mean age at baseline was 61.5 ± 9.0 years, and women accounted for 65.3% of the overall population. During the entire follow-up period, most of the cardiometabolic traits including adiposity index (BMI and waist circumference) and glucolipid profiles showed a modest variation (**Figure 2**). Men have a similar trend of cardiometabolic traits during the entire follow-up period, as compared with women. Age adjusted waist circumference, systolic blood pressure, fasting insulin, HOMA-IR, total cholesterol, and LDL cholesterol experienced the undulation of rising first and then falling. During the first 3-year follow up, age adjusted BMI, diastolic blood pressure, fasting, and OGTT 2-h plasma glucose all showed signs of stabilization, but OGTT 2-h plasma glucose showed increased levels and the other three traits showed decreased levels during the next 3 years. HDL cholesterol showed a continued momentum of decline from baseline, and triglycerides showed a minimum of change during the entire follow-up period.

**Table 1:** Characteristics of participants in the longitudinal study with follow-up at 3 and 6 years, respectively (n = 784).

Characteristics	Baseline	3 years of follow-up	6 years of follow-up	*P* value	SNK test
Age (year)	61.5 ± 9.0	65.1 ± 9.0	67.8 ± 9.0	< 0.0001	C, B, A
Women [n (%)]	512 (65.3)	512 (65.3)	512 (65.3)	/	/
Body mass index (kg/m^2^)	25.0 ± 3.2	25.3 ± 3.2	24.6 ± 3.3	< 0.0001	(B, A), C
Waist circumference (cm)	83.6 ± 8.4	85.2 ± 9.1	83.4 ± 8.8	< 0.0001	B, (A, C)
Current smoking [n (%)]	109 (13.9)	99 (12.6)	78 (10.0)	0.05	/
Current drinking [n (%)]	94 (12.0)	106 (13.5)	105 (13.4)	0.68	/
Systolic BP (mm Hg)	137.5 ± 22.6	142.2 ± 19.7	135.6 ± 19.3	< 0.0001	B, (A, C)
Diastolic BP (mm Hg)	79.5 ± 10.5	80.0 ± 10.2	76.4 ± 9.5	< 0.0001	(B, A), C
Hypertension [n (%)]	416 (53.1)	532 (68.0)	564 (72.0)	< 0.0001	(C, B), A
Lower-BP therapy [n (%)]	246 (31.4)	277 (35.4)	330 (42.1)	0.0001	C, (B, A)
Total cholesterol (mmol/L)	4.8 ± 0.9	5.0 ± 0.8	4.3 ± 1.1	< 0.0001	B, A, C
Triglycerides (mmol/L)	1.3 (0.9, 1.8)	1.2 (0.8, 1.7)	1.3 (0.9, 1.7)	0.17	/
HDL cholesterol (mmol/L)	1.5 ± 0.4	1.4 ± 0.3	1.2 ± 0.4	< 0.0001	A, B, C
LDL cholesterol (mmol/L)	2.7 ± 0.7	3.3 ± 0.8	2.5 ± 0.8	< 0.0001	A, B, C
FPG (mmol/L)	5.6 ± 0.6	5.6 ± 0.9	5.5 ± 0.6	< 0.0001	(B, A), C
OGTT 2-h PPG (mmol/l)	7.0 ± 1.6	7.0 ± 2.1	7.8 ± 2.3	< 0.0001	C, (A, B)
Fasting insulin (pmol/L)	4.9 (2.5, 9.0)	6.9 (4.7, 9.9)	5.7 (4.2, 7.7)	< 0.0001	B, (A, C)
HOMA-IR	1.2 (0.6, 2.3)	1.7 (1.1, 2.7)	1.4 (1.0, 1.9)	< 0.0001	B, (A, C)
HOMA-β	49.5 (23.3, 87.0)	65.1 (44.9, 95.8)	60.1 (42.6, 83.6)	< 0.0001	(B, C), A
RBP4 (ug/mL)	16.4 (12.1, 21.7)	/	/	/	/

Data are presented as mean ± standard deviation (SD), median (interquartile range), or number (percentage). Multiple comparisons were performed by Student-Newman-Keuls (SNK) test. BP, blood pressure; LDL cholesterol, low-density lipoprotein cholesterol; HDL cholesterol, high-density lipoprotein cholesterol; FPG, fasting plasma glucose; OGTT 2-h PPG, oral glucose tolerate test 2-h post load plasma glucose; HOMA-IR, homeostasis model assessment of insulin resistance; HOMA-β, homeostasis model assessment of β cell function; RBP4, retinol binding protein 4; SNK test, Student-Newman-Keuls test. A, B, and C represent baseline, 3-year follow-up, and 6-year follow-up, respectively. Letters within brackets indicate means in different clusters are not significantly different.

The median RBP4 level at baseline was 16.4 (inter-quartile range 12.1-21.7) ug/mL. Notably, women had lower serum RBP4 levels compared with men [15.8 ug/mL (11.3, 21.0) vs. 17.5 ug/mL (13.3, 23.9), P <0.05].

### Association of Serum RBP4 Levels With Adiposity Index and Glucolipid Metabolism Traits

The associations between baseline serum RBP4 levels and adiposity indexes, glucose metabolism traits, and lipid profile are presented in **Table 2**. In the age and sex adjusted model (model 1), baseline serum RBP4 levels were positively associated with BMI, waist circumference, fasting, and OGTT 2-h plasma glucose concentrations, serum triglycerides concentrations, and total cholesterol concentrations. When smoking status and drinking status were further adjusted (model 2), per 1-unit increase in log10-RBP4 was associated with 0.97 kg/m^2^ [95% CI (0.02, 1.93), P=0.046] increment of BMI and 3.12 cm increment of waist circumference [95% CI (0.77, 5.47), P=0.01]. Additional adjustment for BMI, smoking status, and drinking status did not change the associations between baseline serum RBP4 levels and fasting plasma glucose, OGTT 2-h plasma glucose, serum triglycerides, and total cholesterol concentrations. When further adjusted for anti-diabetes drug usage in glucose metabolism traits or lipid-lowering drug usage in the lipid profile (model 3), RBP4 was significantly associated with fasting and 2-h post-loading glucose [β=0.26 (0.05, 0.47), P=0.02, and β=1.70 (1.29, 2.12), P< 0.0001], serum triglycerides [β=0.75, 95% CI (0.54, 0.96), P<0.0001], and total cholesterol [β=0.47, 95% CI (0.23 0.70), P<0.0001]. We did not find any significant associations between serum RBP4 levels and fasting insulin, HOAM-IR, HOMA-β, LDL cholesterol, and HDL cholesterol.

**Table 2:** Adjusted changes (95% CI) in metabolic traits per 1-unit increase in log_10_-RBP4 by using the GEE model.

	* Model 1*		* Model 2*		* Model 3*	
	Unit change (95% CI)	*P* value	Unit change (95% CI)	*P* value	Unit change (95% CI)	*P* value
**Adiposity**						
Body mass index, kg/m^2^	0.98 (0.03, 1.93)	0.04	0.97 (0.02, 1.93)	0.046	/	/
Waist circumference, cm	3.00 (0.64, 5.35)	0.01	3.12 (0.77, 5.47)	0.01	/	/
**Glucose metabolism**						
Fasting plasma glucose, mmol/L	0.26 (0.05, 0.47)	0.01	0.24 (0.03, 0.45)	0.02	0.26 (0.05, 0.47)	0.02
OGTT 2-h plasma glucose, mmol/L	1.64 (1.22, 2.05)	< 0.0001	1.59 (1.16, 2.01)	< 0.0001	1.70 (1.29, 2.12)	< 0.0001
Log_10_ (fasting insulin), pmol/L	-0.01 (-0.14, 0.11)	0.85	-0.06 (-0.22, 0.09)	0.43	-0.07 (-0.23, 0.09)	0.39
Log_10_ (HOMA-IR)	0.03 (-0.07, 0.12)	0.56	-0.01 (-0.12, 0.09)	0.81	-0.02 (-0.13, 0.09)	0.70
Log_10_ (HOMA-β)	0.02 (-0.04, 0.09)	0.48	-0.002 (-0.07, 0.06)	0.97	-0.005 (-0.07, 0.06)	0.93
**Lipids profile**						
Triglycerides, mmol/L	0.78 (0.56, 1.00)	< 0.0001	0.75 (0.54, 0.96)	< 0.0001	0.75 (0.54, 0.96)	< 0.0001
Total cholesterol, mmol/L	0.48 (0.25, 0.71)	< 0.0001	0.45 (0.21, 0.68)	0.0002	0.47 (0.23, 0.70)	< 0.0001
LDL cholesterol, mmol/L	-0.006 (-0.01, 0.0002)	0.06	0.19 (-0.002, 0.37)	0.053	0.02 (-0.03, 0.07)	0.43
HDL cholesterol, mmol/L	-0.04 (-0.13, 0.05)	0.41	-0.01 (-0.1, 0.08)	0.83	-0.02 (-0.10, 0.07)	0.74

Data are changes and 95% confidence intervals (CI) of cardiometabolic traits per 1-unit increase in log10-RBP4. In the generalized estimating equation (GEE) model, the exposure was baseline blood RBP4 levels, the dependent variables were repeated evaluation levels of cardiometabolic traits. Model 1, adjusted for age and sex; model 2, further adjusted for BMI, and smoking and drinking habits based on model 1, while BMI was not adjusted for the adiposity index BMI and waist circumference; model 3, further adjusted for anti-diabetes drug usage based on model 2 for glucose metabolism traits, further adjusted for lipid-lowering drug usage based on model 2 for lipid profile.

To track the change in association between RBP4 and metabolic traits, multiple linear regression analyses were performed at baseline and two follow-up visits. The dynamic *P* values for significance between RBP4 and metabolic traits were presented in **Supplementary Figure S1**.

### Sex-Specific Association of Serum RBP4 Levels With Adiposity Index and Glucolipid Metabolism Traits

**Table 3** shows the sex-specific associations of serum RBP4 levels with adiposity indexes and glucolipid metabolism traits. Baseline serum RBP4 levels (indicated as each 1-unit of log_10_-RBP4) were significantly associated with waist circumference in men [β=4.80, 95% CI (0.43, 9.17), P=0.03] but not in women [β=2.13, 95% CI (-0.57, 4.84), P=0.12] (P for interaction=0.31). Baseline serum RBP4 was significantly associated with OGTT 2-h plasma glucose in women [β=1.99, 95% CI (1.49, 2.50), P <0.0001] but not in men [β=0.61, 95% CI (-0.14, 1.36), P=0.11] (P for interaction=0.001). Both in men and in women, baseline serum RBP4 was significantly associated with serum triglycerides and total cholesterol (both P ≤0.01).

**Table 3:** Adjusted changes (95% CI) in metabolic traits per 1-unit increase in log_10_-RBP4 in participants stratified according to sex by using the GEE model.

	Men (*n *= 272)		Women (*n *= 512)		*P* for interaction
	Change (95% CI)	*P* value	Change (95% CI)	*P* value	
**Adiposity**					
Body mass index, kg/m^2^	0.95 (-0.73, 2.63)	0.27	0.91 (-0.25, 2.07)	0.12	0.93
Waist circumference, cm	4.80 (0.43, 9.17)	0.03	2.13 (-0.57, 4.84)	0.12	0.31
**Glucose metabolism**					
Fasting plasma glucose, mmol/L	0.19 (-0.08, 0.45)	0.16	0.01 (-0.23, 0.25)	0.96	0.52
OGTT 2h plasma glucose, mmol/L	0.61 (-0.14, 1.36)	0.11	1.99 (1.49, 2.50)	< 0.0001	0.001
Log_10_ (fasting insulin), pmol/L	0.03 (-0.09, 0.16)	0.61	-0.09 (-0.28, 0.10)	0.33	0.23
Log_10_ (HOMA-IR)	0.05 (-0.08) 0.19)	0.44	-0.05 (-0.18, 0.09)	0.50	0.25
Log_10_ (HOMA-β)	-0.01 (-0.14, 0.12)	0.84	0.00 (-0.08, 0.08)	0.98	0.91
**Lipids profile**					
Triglycerides, mmol/L	0.49 (0.13, 0.85)	0.007	0.82 (0.56, 1.07)	< 0.0001	0.20
Total cholesterol, mmol/L	0.54 (0.11, 0.97)	0.01	0.45 (0.17, 0.73)	0.002	0.68
LDL cholesterol, mmol/L	0.28 (-0.07, 0.62)	0.12	0.19 (-0.04, 0.42)	0.10	0.65
HDL cholesterol, mmol/L	0.10 (-0.05, 0.26)	0.20	-0.07 (-0.17, 0.04)	0.22	0.07

Data are changes and 95% confidence intervals (CI) of cardiometabolic traits per 1-unit increase in log10-RBP4. In the generalized estimating equation (GEE) model, the exposure was baseline blood RBP4 levels, the dependent variables were repeated evaluation levels of cardiometabolic traits. The adjustments of adiposity index included age, smoking status, drinking status; the adjustments of glucose metabolism traits included age, BMI, smoking status, drinking status, and anti-diabetes drug usage; the adjustments of lipid profile included age, BMI, smoking status, drinking status, and lipid-lowering drug usage. For the interaction test, sex, log10(RBP4), and sex*log10(RBP4) were simultaneously added to the model.

For adiposity indexes, Z score standardized analysis revealed a little bit stronger association of baseline serum RBP4 levels with waist circumference than BMI (**Table 4**). There was a 0.34-units [95% CI (0.07, 0.61), P=0.01] increase in Z score of waist circumference corresponding to a 1-unit increase in log10-RBP4, while a 0.29-units [95% CI (-0.01, 0.59), P=0.051] increase in Z score of BMI. Compared with fasting plasma glucose concentrations, there was a stronger association between baseline serum RBP4 levels and OGTT 2-h plasma glucose concentrations in total participants (**Table 4**). There was only a 0.33-units [95% CI (0.04, 0.62), P=0.03] increase in Z score of fasting plasma glucose concentrations corresponding to a 1-unit increase in log10-RBP4, while a 0.82-units [95% CI (0.62, 1.03), P<0.0001] increase in Z score of OGTT 2-h plasma glucose concentrations (**Table 4**). After stratified by sex, the similar results were found as compared to that in **Table 3**.

**Table 4:** Adjusted changes (95% CI) in Z-score of metabolic traits per 1-unit increase in log10-RBP4 by using the GEE model.

	Total sample (*n *= 784)		Men (*n *= 272)		Women (*n *= 512)		*P* for interaction
	Change in Z-score (95% CI)	*P* value	Change in Z-score (95% CI)	*P* value	Change in Z-score (95% CI)	*P* value	
**Adiposity**							
Body mass index	0.29 (-0.01, 0.59)	0.051	0.30 (-0.24, 0.84)	0.27	0.27 (-0.09, 0.62)	0.14	0.87
Waist circumference	0.34 (0.07, 0.61)	0.01	0.56 (0.05, 1.08)	0.03	0.22 (-0.09, 0.52)	0.17	0.26
**Glucose metabolism**							
Fasting plasma glucose	0.33 (0.04, 0.62)	0.03	0.29 (-0.12, 0.71)	0.16	0.01 (-0.32, 0.33)	0.96	0.34
OGTT 2-h plasma glucose	0.82 (0.62, 1.03)	< 0.0001	0.29 (-0.07, 0.65)	0.11	0.96 (0.72, 1.21)	< 0.0001	0.001
**Lipids profile**							
Triglycerides	0.73 (0.53, 0.94)	< 0.0001	0.40 (0.11, 0.69)	0.007	0.84 (0.58, 1.10)	< 0.0001	0.04
Total cholesterol	0.49 (0.25, 0.74)	< 0.0001	0.59 (0.12, 1.06)	0.01	0.45 (0.17, 0.74)	0.002	0.58

Data are changes and 95% confidence intervals (CI) of Z score transformed cardiometabolic traits per 1-unit increase in log10-RBP4. In the generalized estimating equation (GEE) model, the exposure was baseline blood RBP4 levels, the dependent variables were Z score transformed cardiometabolic traits. The adjustments of adiposity index included age, sex, smoking status, and drinking status; the adjustments of glucose metabolism traits included age, sex, BMI, smoking status, drinking status, and anti-diabetes drug usage; the adjustments of lipid profile included age, sex, BMI, smoking status, drinking status, and lipid-lowering drug usage. For the interaction test, sex, log10(RBP4), and sex*log10(RBP4) were simultaneously added to the model.

## Discussion

In the present study, we systematically assessed the association of baseline RBP4 levels with a longitudinal dynamic cardiometabolic profiles which were evaluated at baseline and two consecutive times of follow-up with 3-years’ interval. We found that in the middle aged and elderly population without diabetes at baseline, baseline higher RBP4 levels were associated with increased BMI, waist circumference, fasting and OGTT 2-h plasma glucose triglyceride, and total cholesterol. Furthermore, sex disparities matter in the relationship of RBP4 with OGTT 2-h plasma glucose, in which RBP4 was associated with OGTT 2-h plasma glucose in women, but not in men.

Elevated circulation RBP4 levels were identified in obese subjects. However, RBP4 levels were associated with BMI in few epidemiological studies. Instead, more studies indicated RBP4 levels were positively associated with visceral body fat or abdominal fat mass (18–21) in individuals with or without diabetes (19) or in healthy women 21 to 67 years old (20). Serum RBP4 was found more highly expressed in visceral fat compared with subcutaneous fat (22), and to be a marker of intra-abdominal fat mass. Similarly, in our present study, we found that RBP4 was significantly associated with a long-term waist circumference level in non-diabetes, a commonly used proxy for visceral fat. Though the change pattern of BMI and waist circumference was similar in that they both increased first at the first 3-years’ follow-up with an average age of 65 years and decreased at the second 3-years’ follow-up with an average age of 68 years, and the effect estimates of waist circumference associated with RBP4 were stronger than that with BMI.

In the current study, we found plasma glucose concentrations in non-diabetes were associated with RBP4 levels as expected. Interestingly, OGTT 2-h plasma glucose concentrations had a more robust association with RBP4 levels than fasting plasma glucose concentrations, which might suggest OGTT 2-h plasma glucose as one main contribution through which increased RBP4 levels confer a higher risk of type 2 diabetes. It has been reported that OGTT 2-h plasma glucose level was regulated by intestine-derived incretin hormone besides glucose-stimulated insulin secretion (23). Intestine-derived incretin hormone can be a potential target for the regulatory role of RBP4. Liraglutide, one of the glucagon-like peptide-1 (GLP-1) analogs that could decrease post-prandial glucose level by regulating incretin in type 2 diabetes, reduced RBP4 gene expression in adipose tissue in mice, which might contribute to the reduction of cardiovascular risk in diabetes (24).

Rare literature reported sexual difference in the association between baseline RBP4 levels and glucose metabolism (14). In the nested case-cohort study from Singapore Chinese, higher risk of type 2 diabetes corresponding to increased plasma RBP4 levels was only observed in women but not in men, the results were still consistent after pooled with two prior studies. In a case-cohort design in which 1080 subjects from the Atherosclerosis Risk in Communities cohort were followed up for 9 years, women in the highest tertile of RBP4 had 74% greater risk of developing diabetes, while no association between RBP4 levels and incident diabetes was found in men (25). The potential mechanisms for the observed sexual difference have not been fully understood. The sex hormone is a possible explanation for the observed sexual differences in association with baseline RBP4 levels and glucose metabolism. On the one hand, RBP4 levels were associated with estrogen levels. A cross-sectional study including 87 Chinese women found a negative association between estrogen levels and RBP4 levels in those who were obese (26). Besides, postmenopausal women have higher RBP4 levels compared to premenopausal women (27). On the other hand, elevated RBP4 levels were reported to be associated with higher free testosterone levels or estimation values of free testosterone in postmenopausal women (26, 27), despite some controversies. It is well established that higher free testosterone is associated with higher risk of disordered glucose metabolism in women, but lower risk in men (28). Therefore, both free testosterone and estrogen are a likely bridge for the sex disparity in glucose regulation.

Sex hormones have pronounced effects on body composition, adipose tissue mass distribution, and metabolic homeostasis. Men have relatively more energy storage in the abdominal and intra-abdominal adipose tissue depots, while premenopausal women store the excess energy in subcutaneous adipose tissue (29, 30). This sex dimorphism may explain the stronger association of the RBP4 level with waist circumference, a simple and practical index for assessing visceral fat.

In this study, there was no association for RBP4 and fasting insulin levels, HOMA-IR, or HOMA-β. These discrepancies within literature may lie in several aspects including that the study populations varied widely in study type, sample size, health condition, and ethnicity. Several studies also declared null association between RBP4 and insulin resistance (31–33). Another more important reason was that the indexes like HOMA-IR and HOMA-β and fasting insulin levels were not adequate to reflect the insulin resistance or β-cell function. In a study including 291 subjects across the spectrum of glycemia, circulating RBP4 levels were significantly and inversely associated with β-cell function indicated by the Stumvoll first-phase and second-phase insulin secretion indices, but not with HOMA-β (34).

RBP4 has also related to an unfavorable lipid metabolism (32, 35–37). But its role in lipid metabolism remains unclear, we know little about what kinds of lipids were regulated and how they were regulated. Most of the previous studies demonstrated that RBP4 was positively associated with triglycerides, while few studies manifested a significant association of RBP4 with other lipids like total cholesterol, low-density lipoprotein cholesterol, and high-density lipoprotein cholesterol (36, 37). The current study presented positive association of RBP4 with triglyceride levels and total cholesterol levels no matter in total population or in sex subgroups, but not with low-density lipoprotein cholesterol and high-density lipoprotein cholesterol. Our results added more evidence for RBP4 and lipid metabolism. Both triglycerides and total cholesterol were positively associated with RBP4, but triglycerides and total cholesterol have different change patterns, total cholesterol decreased significantly while triglycerides remained almost unchanged. In our previous repeated measures study including 1872 participants aged 40 years or older and free of dyslipidemia at baseline, lipid level was measured at baseline and at 4-year follow-up, results showed stable triglyceride levels and increased total cholesterol levels during a 4-year follow-up (38). In another longitudinal study aimed to study changes in participants’ diabetes risk and anthropometrics from baseline to 60 months follow-up, measurements were performed annually up to 60 months after inclusion, both triglycerides and total cholesterol had no significant change (39). However, the result was based on small sample size data, only 120 participants were included in the final analysis. The different study population, sample size, follow-up time, and the usage of statins may be possible explanations for the different change trend in triglycerides and total cholesterol.

The main strength of this study is the longitudinal design with systematic and repeated monitoring for metabolic traits. Systematic metabolic traits including adiposity index, glucose metabolic traits, and blood lipid profile made it practicable to get a comprehensive knowledge about the association of RBP4 on cardiometabolic risk profile. Besides, the repeated measurements of metabolic traits also reduced the bias derived from a single cross-sectional survey. Another strength is the novelty of the current study, as we identified the sexual difference in the association between RBP4 levels and metabolic traits, especially for the 2-h post-loading glucose, which implies an import issue of sex disparities in the disease pathophysiology.

Severe limitations should be acknowledged. First, serum RBP4 was not repeatedly measured at follow-up, which prohibited us to track a long-term trajectory of circulating RBP4 levels. One previous study aimed to assess the biological variation of adipokines RBP4 within individuals by measuring the level of RBP4 twice, approximately 4 months apart (40). The intraclass correlation coefficient for repeated RBP4 measurements was 0.77 (95% CI 0.71, 0.82), indicating excellent reliability. However, the longitudinal changes in blood levels of RBP4 needs further investigation. Second, an imbalanced sex ratio existed in our population. That was partly because women live longer than men, another explanation for that is women retired 5 years earlier than men and have more time to participate in epidemiological investigation. However, we have statistical power to detect the interaction with sex and get the sex-specific conclusions. Third, compared with several previous studies from China (4, 9), the serum RBP4 level in the current study is much lower. Different health conditions of participants is the main explanation for the variance in serum RBP4 level. In the current study, we included non-diabetes participants in relatively good health. Besides, men have higher serum RBP4 levels than women (4, 25), sex distribution difference may be another explanation for the variance in serum RBP4 level. Fourth, we did not test the sex hormone levels for participants in the current study. We could not provide the interaction effects of RBP4 levels with sex hormone levels in the associations with the metabolic changes. Finally, any causal inference could not be established as this was an observational study. Despite its observational nature, this study adds evidence of serum RBP4 with a long-term metabolic profile. Future large scale Mendelian randomization studies or animal models would help to clarify the role of RBP4 in metabolic regulation.

In conclusion, the baseline serum RBP4 level is robustly associated with a longitudinal profile of waist circumference, fasting and OGTT 2-h plasma glucose, triglyceride and total cholesterol, and marginally associated with BMI. The association of RBP4 levels on metabolic traits differ in sex, with stronger association with OGTT 2-h plasma glucose level in women. The results imply an import issue of sex disparities in the disease pathophysiology. Further experimental investigations are required to better understand the molecular mechanisms underlying the sex-specific deterioration of metabolic traits due to elevated RBP4 levels.

## Data Availability Statement

The original contributions presented in the study are included in the article/**Supplementary Material**. Further inquiries can be directed to the corresponding authors.

## Ethics Statement

The studies involving human participants were reviewed and approved by the ethics committee of Ruijin Hospital Affiliated to Shanghai Jiao Tong University School of Medicine. Each patient/participant provided the written informed consent to participate in this study.

## Author Contributions

MX, YB, and JW contributed to the concept and design. JX, HD, YH, and QW contributed to the analysis of data. JX drafted the manuscript. MX edited the manuscript. TW, ML, ZZ, JL, MD, DZ, YX, GN, WW, YB, and MX contributed to collecting and the acquisition of data. All authors made important contributions to critically revising the manuscript for important intellectual content. MX guarantees this work, has full access to the data, and takes responsibility for the integrity of the data. All authors contributed to the article and approved the submitted version.

## Funding

This study was supported by grants from the National Natural Science Foundation of China [grant number 81941017, 81930021, 81970728, 91857205, 82088102, and 81870604], the Shanghai Municipal Education Commission–Gaofeng Clinical Medicine Grant Support [grant number 20152508 Round 2], the Shanghai Shenkang Hospital Development Center [grant number SHDC12019101, SHDC2020CR1001A, and SHDC2020CR3069B]. T.W., M.L., Z.Z., J.L., Y.X., W.W., G.N., J.W., Y.B., and M.X. are members of the Innovative Research Team of High-Level Local Universities in Shanghai.

## Conflict of Interest

The authors declare that the research was conducted in the absence of any commercial or financial relationships that could be construed as a potential conflict of interest.

## Publisher’s Note

All claims expressed in this article are solely those of the authors and do not necessarily represent those of their affiliated organizations, or those of the publisher, the editors and the reviewers. Any product that may be evaluated in this article, or claim that may be made by its manufacturer, is not guaranteed or endorsed by the publisher.
