# Supplementary figures and images for: Sexual Dimorphism in the Association of Serum Retinol-Binding Protein-4 With Long-Term Dynamic Metabolic Profiles in Non-Diabetes
Source: Front Endocrinol (Lausanne). 2022 May 11;13:880467. doi: 10.3389/fendo.2022.880467 (PMC9130550; doi:10.3389/fendo.2022.880467)

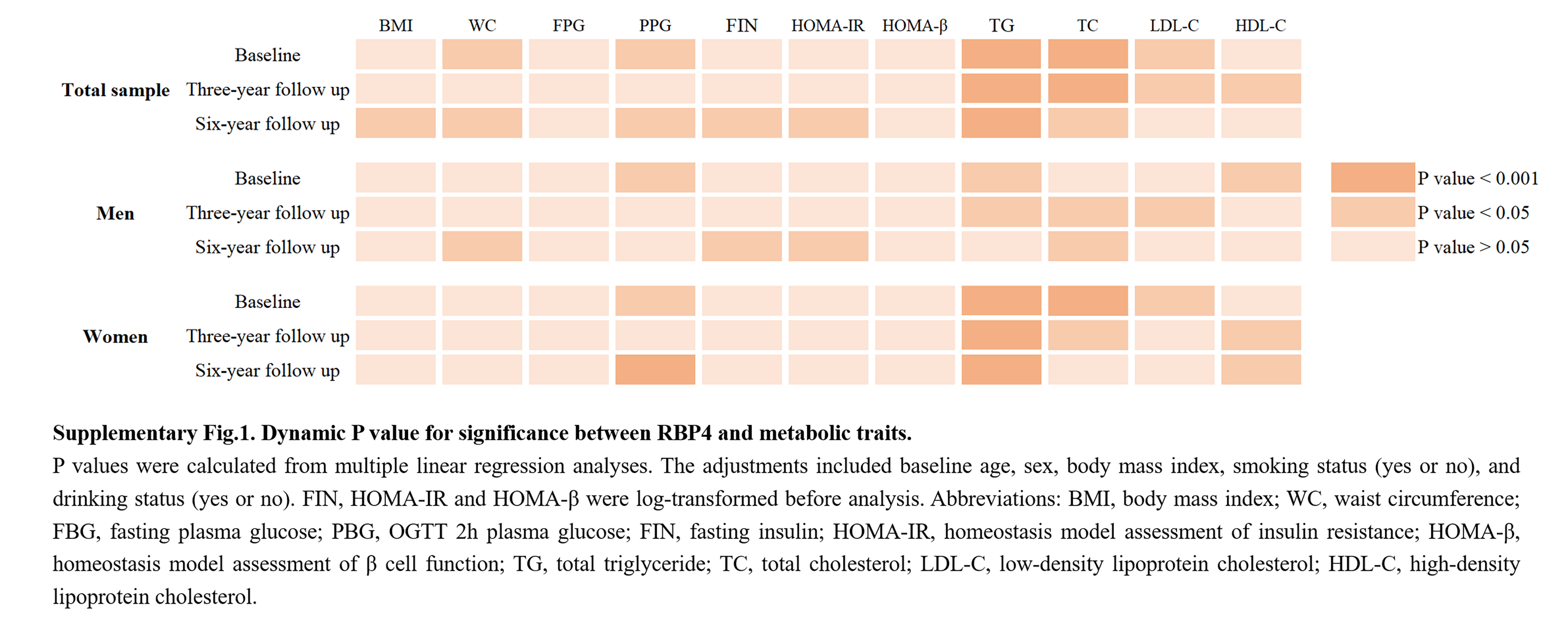

Supplement: Supplementary file 1 [file Image_1.tif]
